# Density Functional Theory Perspective on the Nonlinear Response of Correlated Electrons across Temperature Regimes

**DOI:** 10.1021/acs.jctc.2c00012

**Published:** 2022-04-29

**Authors:** Zhandos Moldabekov, Jan Vorberger, Tobias Dornheim

**Affiliations:** † Center for Advanced Systems Understanding (CASUS), D-02826 Görlitz, Germany; ‡ Helmholtz-Zentrum Dresden-Rossendorf (HZDR), D-01328 Dresden, Germany

## Abstract

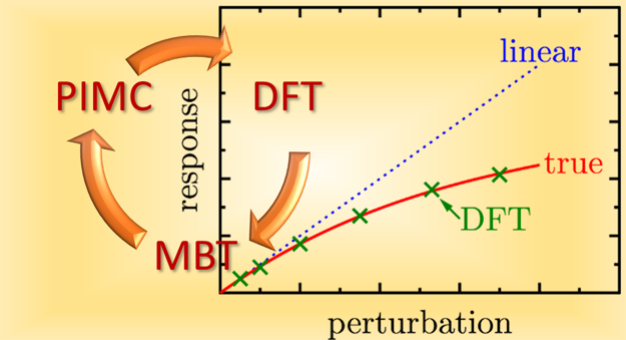

We explore a new formalism to study the nonlinear electronic density response based on Kohn–Sham density functional theory (KS-DFT) at partially and strongly quantum degenerate regimes. It is demonstrated that the KS-DFT calculations are able to accurately reproduce the available path integral Monte Carlo simulation results at temperatures relevant for warm dense matter research. The existing analytical results for the quadratic and cubic response functions are rigorously tested. It is demonstrated that the analytical results for the quadratic response function closely agree with the KS-DFT data. Furthermore, the performed analysis reveals that currently available analytical formulas for the cubic response function are not able to describe simulation results, neither qualitatively nor quantitatively, at small wavenumbers *q* < 2*q*_F_, with *q*_F_ being the Fermi wavenumber. The results show that KS-DFT can be used to describe warm dense matter that is strongly perturbed by an external field with remarkable accuracy. Furthermore, it is demonstrated that KS-DFT constitutes a valuable tool to guide the development of the nonlinear response theory of correlated quantum electrons from ambient to extreme conditions. This opens up new avenues to study nonlinear effects in a gamut of different contexts at conditions that cannot be accessed with previously used path integral Monte Carlo methods.

## 1 Introduction

Quantum linear response theory (LRT) has been actively developed since the formulation of the foundations of quantum mechanics and has become one of the most fundamental theories for the computation of various properties.^1^ At the same time, the ongoing development of technological and, along with it, experimental capabilities has resulted in the need for a theory that captures phenomena beyond the linear response regime. Specific examples include plasmonics,^2,3^ optics,^4,5^ and more recently warm dense matter (WDM)^6,7^—an extreme state that occurs in astrophysical objects^8,9^ and that is also relevant for technological applications.^10−13^

However, in contrast to the LRT, the foundations of a quantum theory of the nonlinear response (NLRT) at finite wavenumbers is far from being established even for simple model systems such as a free electron gas.^14,15^ In this regard, the lack of a reliable theoretical foundation makes the ab initio simulation an indispensable tool to guide the development of the NLRT. This was demonstrated recently for WDM by performing path integral quantum Monte Carlo (PIMC) simulations.^16,17^ However, while these results are exact, the fermion sign problem^18,19^ limits their application to moderate levels of quantum degeneracy. In contrast, the thermal Kohn–Sham density functional theory (KS-DFT) method^20^ does not suffer from this limitation. Indeed, it has become standard practice to study the linear electronic response^21,22^ based on the KS orbitals. In this work, we explore a new KS-DFT based approach to the nonlinear electronic response of arbitrary materials. First, this methodology allows us to compute higher-order (meaning quadratic, cubic, etc. with respect to the perturbation amplitude) response functions, with the only approximation being given by the choice of the exchange–correlation (XC) functional. In addition, we can straightforwardly estimate the validity range of LRT, which is highly important in its own right.

As a particular example, we apply this approach to the free electron gas^14,15^—the archetypical model system with general relevance for numerous applications in condensate matter physics and high-energy-density science. From a many-particle physics perspective, we note that it is imperative to first develop a NLRT for this general free electron gas model, before applying the NLRT to specific cases.

In this context, thermal KS-DFT^20^ constitutes the method of choice because it allows calculations over large temperature ranges covering the strongly to partially degenerate regimes. Moreover, we note that the general nature of our present NLRT approach makes it directly useable for high-*T* DFT methods,^23,24^ including orbital-free formulations.^25^ Since the free electron gas model and the NLRT have important applications in WDM,^6,26^ we start from relatively high temperatures relevant for laboratory astrophysics^27−29^ as well as astrophysical models,^9^ inertial confinement fusion,^10^ and the synthesis of new materials at extreme conditions.^11−13^ At these parameters, we can benchmark KS-DFT results against available PIMC results.^6,16,17^ In addition, we consider lower temperatures down to the electronic ground state that are relevant for condensed matter physics.

It is convention to give the temperature *T* and density *n*_0_ of the free electron gas using the reduced temperature θ = *T*/*T*_F_ (with *T*_F_ being the Fermi temperature) and the mean inter-particle distance in a.u., *r*_s_ = (4π*n*_0_/3)^1/3^. For example, a rather loose definition of the WDM regime corresponds to temperatures 0.1 ≲ θ ≲ 10 and densities 0.5 ≲ *r*_s_ ≲ 10.

The prospect of the observation of nonlinear phenomena in WDM has triggered an active investigation of the nonlinear density response properties of the free electron gas by Dornheim et al.^16,17,30^ using the ab initio PIMC method.^18,31^ The focus of these PIMC studies was the static density response of WDM at temperatures θ ≥ 1. The main reason for not considering lower temperatures was the aforementioned fermion sign problem,^18^ which results in an exponential increase in the computation time with decreasing temperature. Although there are other quantum Monte Carlo (QMC) methods that have different domains of applicability, such as the configuration PIMC approach,^32,33^ the permutation blocking PIMC method,^34,35^ and a phaseless auxiliary-field QMC technique,^36^ there are always parameters at which QMC methods encounter significant difficulties. Approximately, the problematic domain for QMC methods corresponds to θ < 1 and *r*_s_ ≳ 2.^33,36,37^

On the other hand, the parameter range corresponding to densities *r*_s_ ≳ 2 and temperatures 0.01 ≲θ < 1 is highly important for numerous applications. Recently, it was shown that the static nonlinear density response functions of the electron gas can be used for the construction of advanced kinetic energy functionals required for orbital-free density functional theory (OF-DFT)-based simulations^38,39^ with applications at ambient^40^ and extreme conditions.^41,42^ Additionally, nonlinear density response functions can extend quantum fluid models (quantum hydrodynamics and time-dependent OF-DFT) beyond the weak perturbation regime.^43−48^ Moreover, static nonlinear density response functions are needed for the systematic improvement of effective pair interaction models for WDM^49−52^ and liquid metals.^53−55^ Finally, Moldabekov and co-workers^56,57^ have recently suggested to deliberately probe the nonlinear regime in X-ray Thomson scattering experiments^58^ as an improved method for the inference of plasma parameters such as the electronic temperature. However, these applications remain in their infancy since the NLRT of correlated electrons is significantly less developed compared to the LRT.^37^ One of the reasons is that the derivations in the NLRT are much more mathematically involved.^59−61^ In fact, the NLRT is burdened with easy-to-make-mistake mathematical tasks and poorly converging integrals. Therefore, the ab initio calculation of the NLRT properties across parameter ranges is required not only to describe certain phenomena but also to guide and test new theoretical developments.

The key goal of this work is to demonstrate the high value of KS-DFT to study the nonlinear density response across temperature regimes as an alternative to much more expensive—for certain parameters even prohibitively expensive—QMC simulations. This is achieved by developing and testing the KS-DFT based methodology for the analysis and investigation of the higher order static density response functions. Therefore, first of all, we show that KS-DFT can be effectively used to compute static nonlinear density response properties of correlated electrons at low temperatures (θ ≲ 1) and is able to reproduce available PIMC results at θ = 1. Second, we provide an analysis of the available theoretical results for the diagonal parts of quadratic and cubic response functions by combining the KS-DFT simulation of correlated electrons, the KS-DFT calculations with the XC functional set to zero, and recently developed machine learning (ML) representation of the local field correction (LFC) of the free electron gas.^62,63^ This confirms the high accuracy of the analytical results for the quadratic response function and reveals the significant deficiency of the available analytical results for the cubic response function. Finally, we are able to show the change in the characteristic features of the NLRT functions on the way from moderately to strongly degenerate regimes.

The paper is organized as follows: in Section 2, we provide the theoretical background of the studied NLRT characteristics; in Section 3, we give the description of the performed simulations; the new results are presented and discussed in Section 4; and the paper is concluded by summarizing the main findings and providing an outlook over future investigations in Section 5.

## 2 Theory

First, we briefly discuss the state-of-the-art theory of the static nonlinear density response functions. Along with that, we establish the terminology used throughout the paper. In general, the definition of the NLRT functions follow from the perturbative expansion of the density *n*(**r**) around its unperturbed value *n*_0_(**r**).^16,59,60^ Specifically, we consider the response of the uniform electron gas to an external harmonic perturbation,^6,64^*V*(**r**) = 2*A*cos(**q**·**r**), with amplitude *A* and wavenumber **q**. In this case, the Fourier expansion of the density distribution reads^16^
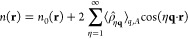
1where we have introduced the density perturbation components in Fourier space ⟨ρ̂_**k**_⟩_*q*,*A*_. The latter quantity is essentially the density perturbation in **k** space induced by an external perturbation with amplitude *A* and wavenumber **q**.

From eq 1, we see that ⟨ρ̂_**k**_⟩_*q*,*A*_ has non-zero components at multiples of the perturbing field wavenumber, that is, at *k* = η**q** with η being an integer number. We refer to ⟨ρ̂_η**q**_⟩_*q*,*A*_ at η = 1, η = 2, and η = 3 as density perturbations at the first, second, and third harmonics, respectively. Next, using the density response ⟨ρ̂_**k**_⟩_*q*,*A*_, we arrive at the following definitions of the density response functions^16,64,65^
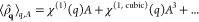
2
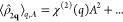
3
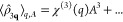
4where χ^(1)^ (*q*) is the linear response function, χ^(1,cubic)^ (*q*) is the cubic response function at the first harmonic, χ^(2)^ (*q*) is the quadratic response function, and χ^(3)^ (*q*) is the cubic response function at the third harmonic. Evidently, eqs 2–4 are given by expansions in terms of the perturbation amplitude *A* and are accurate up to the third order. While the density response is only given by a single term at the wavenumber of the original perturbation within LRT, the consideration of nonlinear effects leads to a richer picture including the excitation of higher-order harmonics.

In the ideal Fermi gas approximation, the linear response function χ_0_^(1)^ is given by the (temperature-dependent) Lindhard function.^66^ On the same level of description, Mikhailov expressed the ideal quadratic response function χ_0_^(2)^ (*q*) and ideal cubic response function at the third harmonic χ_0_^(3)^ (*q*) in terms of the Lindhard function^65,67^
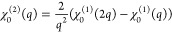
5
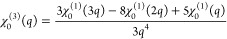
6

Next, on the mean-field level, usually called RPA, the results for χ^(2)^ (*q*) and χ^(3)^ (*q*) can be obtained by taking into account screening on the level of the linear response and dropping quadratic or higher order corrections to screening^16^
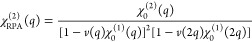
7and
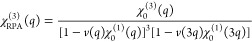
8

Finally, some electronic correlation effects beyond the mean-field level can be taken into account using a LFC *G*(*k*) in the denominator^16^
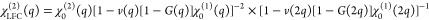
9and
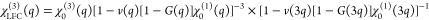
10Similarly, the screened eqs 7–10 take into account electronic XC on the basis of the LRT. However, contrary to the case of the LFC in the linear response function, the insertion of the LFC here cannot give an exact result as further terms are missing. Equations 9 and 10 are easy-to-compute solutions for the case of a harmonically perturbed electron gas since the static LFC at the parameters of interest is readily available from a ML representation, which is based on QMC simulation results.^62,63^

Finally, we note that there are no satisfactory analytical results for the ideal cubic response function at the first harmonic χ_0_^(1,cubic)^ (*q*). Nevertheless, there is a formal relation between χ_0_^(1,cubic)^ and the cubic response function of correlated electrons, which follows from perturbative analysis based on the Green function method^16^
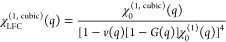
11

Note that the mean-field result for the cubic response function at the first harmonic follows from eq 11 by setting *G* = 0
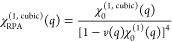
12

In this work, we use the KS-DFT method to compute the set of density response functions defined by eqs 2–4 and subsequently verify the quality of the KS-DFT results by comparing with PIMC results at θ = 1. Then, we use KS-DFT results to analyze the analytical approximations given by eqs 5–10 in the wide range of parameters inaccessible for QMC methods. This allows us to unambiguously assess the importance of the negligible higher order (nonlinear) screening and LFC effects.

## 3 Simulation Details

The computational workflow consists of four main steps: First, the thermal KS-DFT simulations^20^ of the free electron gas perturbed by an external field *V*(**r**) = 2*A*cos(**q·r**) are performed for different *A* and **q** values; second, the wave functions from KS-DFT simulations are used to compute the total density distribution along the direction of the wave vector **q**; third, the density perturbation components in *k* space ⟨ρ̂_**k**_⟩_*q*,*A*_ are computed using eq 1; and finally, the density response functions are found by fitting data for ⟨ρ̂_**k**_⟩_*q*,*A*_ using eqs 2–4.

To begin with, we consider a strongly correlated electron gas with *r*_s_ = 6 at θ = 1 and *r*_s_ = 5 at θ = 0.01. At *r*_s_ = 6, we compare the results with the available finite temperature PIMC data for the linear and nonlinear density response functions.^16^ At *r*_s_ = 5, we compare with diffusion quantum Monte Carlo (DMC) results for the linear density response function computed by Moroni, Ceperley, and Senatore.^64^ Furthermore, we investigate a metallic density with *r*_s_ = 2 at three different values of the degeneracy parameter, θ = 1, θ = 0.5, and θ = 0.01. In this case, we also benchmark results against PIMC data at θ = 1 and compare with the linear density response function from the DMC simulations at θ = 0.01.

The KS-DFT simulations of the free electron gas were performed using the GPAW code,^68−71^ which is a real-space implementation of the projector augmented-wave method. The number of particles in the main simulation box varied in the range from 14 to 66. Accordingly, the main cubic cell size is defined by *r*_s_ and *N* as *L* = *r*_s_(4/3π*N*)^1/3^. The direction of the perturbation is set to be along the *z*-axis. Due to periodic boundary conditions, the value of the perturbation wavenumber of the external harmonic field is defined by the reciprocal lattice vectors of the main simulation cell *q* = η × 2π/*L*, with η being a positive integer number. We used a Monkhorst-Pack^72^ sampling of the Brillouin zone with a *k*-point grid of *N*_k_ × *N*_k_ × *N*_k_ total points, with *N*_k_ = 12 at *r*_s_ = 2 and *N*_k_ = 8 at *r*_s_ = 6 and *r*_s_ = 5. The calculations were performed using a plane-wave basis where the cutoff energy has been converged to 800 eV at θ = 1 and *r*_s_ = 2 and to 440 eV at the rest of the *r*_s_ and θ values. The number of orbitals is set to *N*_b_ = 500 at *r*_s_ = 2 and θ = 1 with the smallest occupation number *f*_min_ ≲ 10^–7^. We set *N*_b_ = 240 at *r*_s_ = 6 and θ = 1 and *N*_b_ = 140 at *r*_s_ = 2 and θ = 0.5 (*f*_min_ ≲ 10^–6^). At θ = 0.01, we set *N*_b_ = 70 for *N* = 66 particles and *N*_b_ = 2 *N* for *N* = 20 and *N* = 14 particles (with *f*_min_ = 0).

At *r*_s_ = 2, the perturbation amplitudes are set in the range from *A* = 0.01 to *A* = 0.1 with a step of Δ*A* = 0.03 (here, *A* is in Hartree atomic units). At *r*_s_ = 6 and *r*_s_ = 5, the perturbation amplitudes are in the range from *A* = 0.002 to *A* = 0.017 with the step Δ*A* = 0.005. These values of the perturbation amplitudes used for the calculation of the density response functions were found empirically guided by the requirement Δ*n*/*n*_0_ ≪ 1 and by testing the validity of eqs 1–4. Examples of the dependence of the density perturbation ⟨ρ̂_**k**_⟩_*q*,*A*_ on *A* and the application of eqs 1–4 are illustrated in the Appendix.

The XC functional in our KS-DFT simulations is the local density approximation (LDA) in the Perdew–Zunger parametrization.^73^ Recently, it was demonstrated for θ = 1 that commonly used GGA functionals such as PBE,^74^ PBEsol,^75^ AM05,^76^ and the meta-GGA functional SCAN^77^ are not able to provide a superior description compared to LDA in the case of the free electron gas perturbed by an external field with fixed wavenumber **q** when Δ*n*/*n*_0_ < 1.^78,79^ We do not aim to further study this problem in this work. Therefore, we do not consider other types of XC functionals beyond LDA.

In addition to the LDA-based calculations, we performed simulations with zero XC functional [to which we refer to as DFT (RPA)]. This allows us to obtain exact results for the density response on the mean-field level. The value of this type of KS-DFT calculations allows us to assess the accuracy of the corresponding theoretical mean-field expressions given in eqs 7 and 8. Furthermore, once the analytical results have been verified, the KS-DFT calculations on the mean-field level can be combined with the LFC to compute a highly accurate response function. We demonstrate that this is the case for the quadratic response function and the cubic response function at the first harmonic using eqs 9 and 11.

## 4 Results

### 4.1 Strongly Correlated Hot Electrons

We start the discussion of our simulation results by considering the strongly correlated electron gas with the density parameter *r*_s_ = 6 and at the reduced temperature θ = 1. This corresponds to WDM generated in evaporation experiments.^80^ At these parameters, we can benchmark the KS-DFT calculations against previous PIMC calculations.^6,16^

#### 4.1.1 Linear Density Response in the WDM Regime

First, we verify that our KS-DFT calculations provide accurate data for the linear static density response function χ^(1)^ (*q*), which allows us to systematically analyze and exclude the possibility of finite size effects.^81^ We start this analysis by comparing the linear static density response function computed using KS-DFT with the exact analytical results on the mean-field level and with χ^(1)^ of the correlated electron gas computed using the exact data for the LFC.^62^

**Figure 1:**
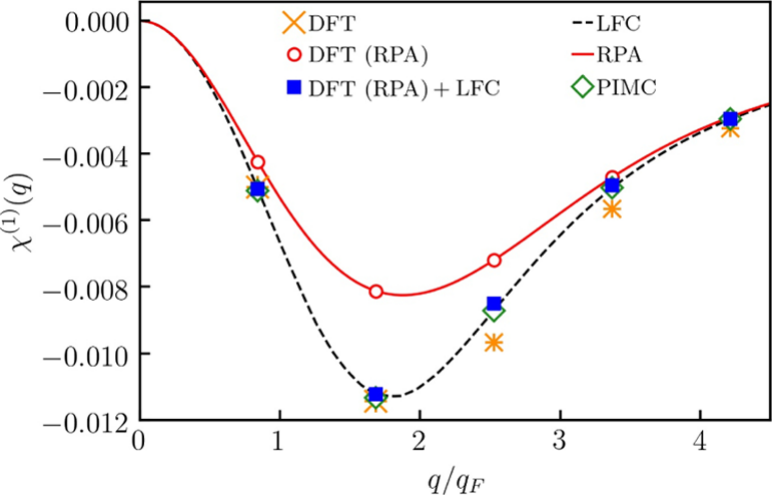
Linear static density response function at *r*_s_ = 6 and θ = 1.

In Figure 1, we present the KS-DFT results computed using *N* = 14 electrons. In this case, the cell size is *L* = 12.335 Å, and the accessible values of the wavenumbers are multiples of *q*_min_ ≃ 0.8427*q*_F_.

From Figure 1, first of all, we see that the linear density response function computed using KS-DFT with zero XC functional, χ̃_RPA_^(1)^, accurately reproduces the exact random phase approximation (RPA) result for the static linear response function,
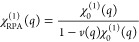
13where *v*(*q*) = 4π/*q*^2^ and χ_0_^(1)^ (*q*) is the Lindhard function. This shows that finite size effects in our KS-DFT calculations with as few as 14 electrons is negligible in the considered case. For completeness, we note that this is consistent with previous findings of PIMC simulations at similar conditions.^62^

Second, we combine the density response function computed using the KS-DFT with zero XC functional, χ̃_RPA_^(1)^, with the LFC to find the linear density response function of correlated electrons
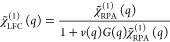
14where *G*(*q*) is computed using the ML representation of the LFC.^62^

From Figure 1, we see that χ̃_LFC_^(1)^ (*q*) [labeled as DFT(RPA) + LFC] is in excellent agreement with the exact value computed using the Lindhard function
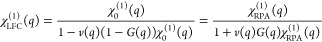
15where after the second equality, we used eq 13 to express χ_LFC_^(1)^ (*q*) in terms of χ_RPA_^(1)^ (*q*).

Note that eq 14 follows from eq 15 after the substitution χ̃_RPA_^(1)^ → χ_RPA_^(1)^ and χ̃_LFC_^(1)^ → χ_LFC_^(1)^.

Now, after verifying that our calculations are not affected by the finite size effect, we compare the LDA-based KS-DFT calculations of the linear density response function, χ̃_LDA_^(1)^ (*q*), with the exact result χ_LFC_^(1)^ (*q*). From Figure 1, we see that χ̃_LDA_^(1)^ (*q*) is in good agreement with χ_LFC_^(1)^ (*q*) at *q* < 2*q*_F_ (with *q*_F_ being the Fermi wavenumber) and exhibits significant disagreements at *q* > 2*q*_F_. To understand this finding, we recall that the LDA corresponds to the long wavelength approximation of the LFC with *G*_LDA_ = γ*k*^2^, where γ is defined by the compressibility sum rule.^82^ This approximation is applicable at *q* ≲ *q*_F_ and increasingly deviates from the exact result with the increase in the wavenumber beyond 2*q*_F_.^78^ Note that all χ̃_LDA_^(1)^ (*q*), χ̃_LFC_^(1)^ (*q*), and χ_LFC_^(1)^ (*q*) tend to χ_RPA_^(1)^ (*q*) in the limit of large wavenumbers since the screening factor dominates over XC effects in this limit. This can be seen from eqs 14 and 15, where the LFC is suppressed by the factor *q*^–2^.

The insights that we have gained considering χ̃_RPA_^(1)^, χ̃^(1)^ (*q*), and χ̃_LDA_(*q*) will help us understand the KS-DFT results for the higher-order nonlinear density response functions discussed in the next subsection. Furthermore, we use a tilde over a symbol to differentiate response functions calculated using KS-DFT from the theoretical definitions.

#### 4.1.2 Nonlinear Density Response in the WDM Regime

**Figure 2:**
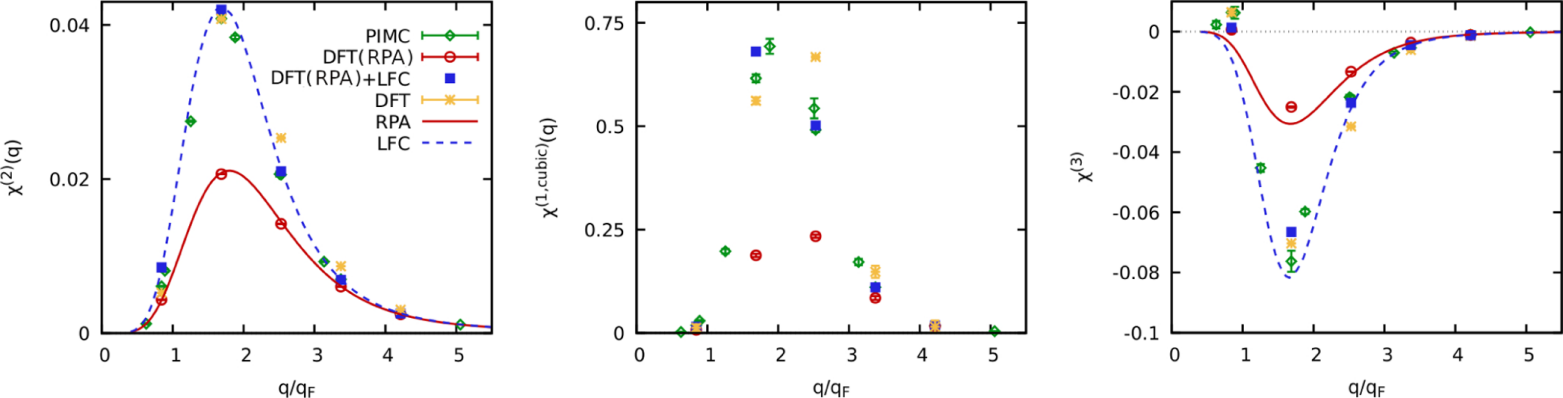
Nonlinear static density response functions at *r*_s_ = 6 and θ = 1. Left: quadratic response function at the second harmonic. Middle: cubic response function at the first harmonic. Right: cubic response function at the third harmonic. We note that analytical results for the cubic response function at the first harmonic are presently not available.

Our new results for the nonlinear density response functions at *r*_s_ = 6 and θ = 1 are presented in Figure 2. In particular, the left panel shows the results for the quadratic response function (defined by eq 3), the middle panel presents data for the cubic response function at the first harmonic (the cubic term in eq 2), and the right panel shows the results for the cubic response function at the third harmonic (defined by eq 3).

First of all, we observe that the LDA XC functional-based calculations are generally in good agreement with the PIMC results at *q* < 2*q*_F_ and overestimate the considered nonlinear density response functions at *q* > 2*q*_F_. The reason for this behavior of the LDA-based calculations is the inaccuracy of the LFC incorporated in the LDA at *q* > 2*q*_F_ as it has been discussed in Section 4.1.1 above.

Next, from the left panel of Figure 2, we see that KS-DFT calculations with XC set to zero [i.e. DFT(RPA)] are in excellent agreement with the theoretical RPA curve for the quadratic response function. This confirms the high quality of the analytical result eq 7 in the WDM regime. In the case of the cubic response function at the third harmonic, as shown in the rightmost panel of Figure 2, we observe that eq 8 is accurate at *q* > 2*q*_F_ but overestimates the response at *q* < 2*q*_F_. Note that the LDA-based KS-DFT results, the KS-DFT calculations without XC, DFT(RPA), and the PIMC results all have positive sign at *q* < *q*_F_, while the theoretical curves fail to capture the change in the sign of the cubic response function at the third harmonic with decrease in wavenumber.

Let us next combine the KS-DFT data for the quadratic response computed with zero XC functional, χ̃_RPA_^(2)^ (*q*), with the LFC. For that, we express χ_LFC_^(2)^ (*q*) via χ_RPA_^(2)^ (*q*) using eqs 7 and 9. Then, we perform substitutions χ̃_LFC_^(2)^ (*q*) → χ_LFC_^(2)^ (*q*) and χ̃_RPA_^(2)^ (*q*) → χ_RPA_^(2)^ (*q*). As the result, we have the following relation
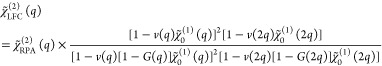
16

where χ̃_0_^(1)^ (*q*) can be extracted from χ̃_RPA_^(1)^ (*q*) as
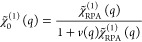
17

Comparing the results calculated using eq 16 with the PIMC data, we conclude that the relationship (eq 9) is fulfilled with high accuracy.

Similarly, we derive the connection between χ̃_LFC_^(1,cubic)^ (*q*) and χ̃_RPA_^(1,cubic)^ (*q*) using eqs 11 and 12 and replacing χ_LFC_^(1,cubic)^ (*q*) → χ̃_LFC_^(1,cubic)^ (*q*) and χ_RPA_^(1,cubic)^ (*q*) → χ̃_RPA_^(1,cubic)^ (*q*). As the result, we find
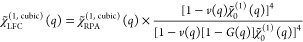
18

Using χ̃_RPA_^(1,cubic)^ (*q*) obtained from KS-DFT simulations with zero XC and the LFC computed using the ML representation,^62^ we have found that eq 18 reproduces the PIMC results in the entire range of the wavenumbers as it can be seen from the middle panel of Figure 2. It is only the availability of χ̃_RPA_^(1,cubic)^ (*q*) that allows us to estimate the cubic response at the first harmonic with PIMC accuracy as no analytical theory for χ_0_^(1,cubic)^ currently exists.

To further explore the combination of the KS-DFT calculations with zero XC functional and the ML representation of the LFC, we next analyze the quality of the theoretical result (eq 10) for the cubic response at the third harmonic. Using eqs 8 and 10 and replacing χ_LFC_^(3)^ (*q*) by χ̃_LFC_^(3)^ (*q*) and χ_RPA_^(3)^ (*q*) by χ̃_RPA_^(3)^ (*q*), we arrive at the following relation between χ_RPA_^(3)^ (*q*) computed using the KS-DFT calculations with zero XC functional and the LFC
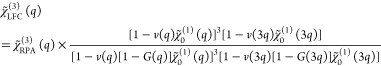
19

The comparison of χ̃_LFC_^(3)^ (*q*) with the PIMC results is presented in the right panel of Figure 2. From this figure, we see that χ̃_LFC_^(3)^ (*q*) significantly deviates from the PIMC data at *q* < 2*q*_F_. This means that the relation (eq 10) does not provide an adequate description of the correlated electron gas. This is expected since we have already demonstrated above that the RPA result (eq 8) is inadequate at *q* < 2*q*_F_. Therefore, the description of the screening on the mean-field level must first be improved to describe the actual system.

### 4.2 Strongly Correlated and Strongly Degenerate Electrons

Next, we investigate the strongly degenerate case with *r*_s_ = 5 and θ = 0.01. In this regime, we are able to verify our KS-DFT calculations by comparing with the accurate DMC calculations of the linear static density response function, χ^(1)^, by Moroni, Ceperley, and Senatore.^64^

Additionally, we further assess possible finite size effects at low temperature by comparing the simulation results for *N* = 14 particles to the results computed using *N* = 20, *N* = 38, and *N* = 66 particles. In this case, the cell size is *L* = 10.28 Å (for *N* = 14), *L* = 11.577 Å (for *N* = 20), *L* = 14.34 Å (for *N* = 38), and *L* = 17.236 Å (for *N* = 66). Correspondingly, the accessible values of the wavenumbers are multiples of *q*_min_ ≃ 0.8427*q*_F_ (for *N* = 14), *q*_min_ ≃ 0.74822*q*_F_ (for *N* = 20), *q*_min_ ≃ 0.604*q*_F_ (for *N* = 38), and *q*_min_ ≃ 0.50*q*_F_ (for *N* = 66).

#### 4.2.1 Linear Density Response in the Limit of Strong Degeneracy

**Figure 3:**
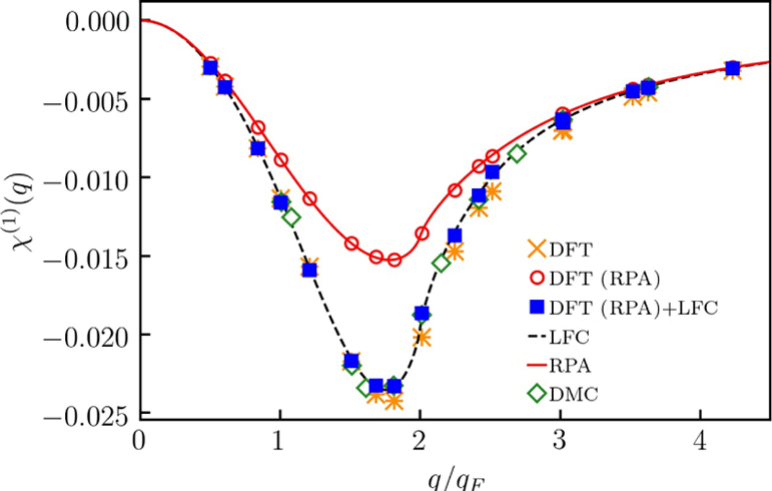
Comparison of the DMC data by Moroni, Ceperley, and Senatore^64^ with DFT results for the linear response function at *r*_s_ = 5 and θ = 0.01.

In Figure 3, we present results for the static linear density response function. Evidently, the results for χ̃_RPA_^(1)^ computed using different numbers of particles accurately reproduces the exact mean-field level result χ_RPA_^(1)^, eq 13. Furthermore, at all considered numbers of particles, the combination of χ̃_RPA_^(1)^ with the LFC using eq 14 allows us to reproduce the exact result given by eq 15. Therefore, the reduction of the number of particles from *N* = 66 to *N* = 38, then to *N* = 20, and further to *N* = 14 does not lead to a deterioration of the quality of the data for χ̃_RPA_^(1)^. This confirms the remarkable convergence of the KS-DFT simulations for as few as *N* = 14 particles.

To get a picture about the quality of the LDA-based calculations, we compare χ̃_LDA_^(1)^ with the DMC results by Moroni et al.^64^ and with χ_LFC_^(1)^ computed using the ML representation of the LFC by Dornheim et al.^62^ Despite the fact that the LDA is designed to describe only the long wavelength limit of the LFC, we observe that in the strongly degenerate case, the LDA-based KS-DFT calculations provide high-quality results for the linear density response function with a level of accuracy similar to the ground-state QMC calculations.

#### 4.2.2 Nonlinear Density Response in the Limit of Strong Degeneracy

**Figure 4:**
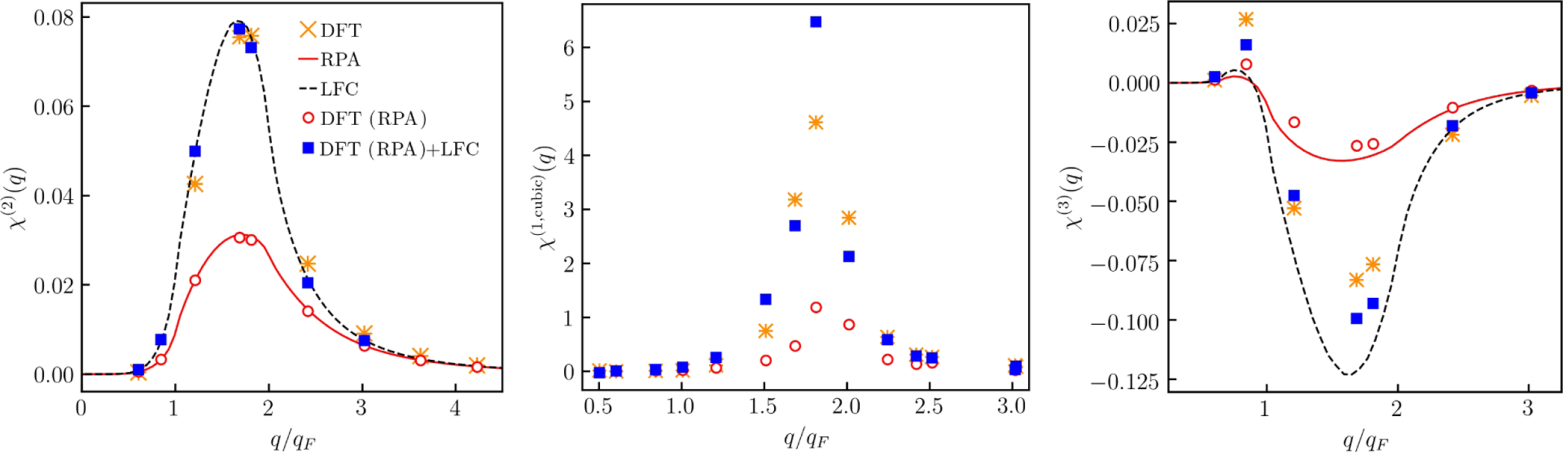
NLRT functions at *r*_s_ = 5 and θ = 0.01. Left: quadratic response function at the second harmonic. Middle: cubic response function at the first harmonic. Right: cubic response function at the third harmonic. We note that analytical results for the cubic response function at the first harmonic are presently not available.

After successfully testing the accuracy of our KS-DFT simulations on the linear density response function, we analyze results for the higher order density response functions presented in Figure 4. In the left panel, we see that χ̃_RPA_^(2)^ is in excellent agreement with χ_RPA_^(2)^ and that χ̃_LFC_^(2)^ also reproduces χ_LFC_^(2)^. This confirms the correctness of the analytical results for the quadratic response function given by eqs 7 and 9 in the limit of strong degeneracy. The LDA-based data χ̃_LDA_^(2)^ provides an adequate description of the quadratic response function and captures the effect of the stronger response when XC effects are included compared to the mean-field level results χ_RPA_^(2)^ and χ̃_RPA_^(2)^. Certain quantitative disagreements between χ̃_LDA_^(2)^ and χ_LFC_^(2)^ can be understood by noting that accurate data for LFC (beyond LDA) is needed to correctly describe the quadratic response. In fact, Dornheim et al.^57^ recently pointed out that the quadratic response is directly related to three-body correlations, which explains this sensitivity to XC effects.

The middle panel of Figure 4 presents data for the cubic response at the first harmonic. Compared to the partially degenerate case with θ = 1, the results exhibit a much sharper peak and much stronger response at 1.5*q*_F_ < *q* < 2*q*_F_. At these wavenumbers, the difference between χ_LFC_^(1,cubic)^ and χ̃_LDA_^(1,cubic)^ are most likely a direct consequence of the fact that the cubic response depends on the fourth power of the LFC, meaning that any deviations from the correct response in the first order gets amplified when a higher-order response is considered.

The right panel of Figure 4 shows the cubic response at the third harmonic. In this case, the theoretical result for the response at the mean-field level given by eq 8, χ_RPA_^(3)^, fails to reproduce the exact data χ̃_RPA_^(3)^. This extends the conclusion that eq 8 fails to correctly describe the screened response from the WDM regime considered earlier to the case of strong degeneracy. As a consequence, being built upon eq 8, the LFC result χ_LFC_^(3)^ defined by eq 10 also does not provide the correct description of the cubic response of the correlated electron gas at the third harmonic. This makes the analysis based on the comparison of χ̃_LFC_^(3)^ and χ̃_LDA_^(3)^ less meaningful. Since the LDA is an approximation to the true XC effects, χ̃_LDA_^(3)^ cannot be considered to be the exact result. Nevertheless, it provides the correct quantitative outcome. Particularly, we see from the right panel of Figure 4 that XC effects lead to a stronger response of the system compared to χ_RPA_^(3)^. We stress that χ̃_RPA_^(3)^ is still the exact ab initio result for the cubic response on the mean-field level. Therefore, it can be used to verify theoretical derivations. Once a more accurate theoretical result for χ_RPA_^(3)^ that includes nonlinear screening effects that are negligible in eq 8 is derived, the correct way to include the LFC should directly follow.

### 4.3 Free Electron Gas at Metallic Density

As a particularly important regime from the point of view of applications, we next consider *r*_s_ = 2, which is a characteristic metallic density. In this case, we investigate three different values of the degeneracy parameter, namely, θ = 1, θ = 0.5, and θ = 0.01. For θ = 1, we have performed series of calculations with *N* = 14, *N* = 20, and *N* = 34. At θ = 0.5, we have considered *N* = 14 and *N* = 34 particles. At θ = 0.01, we have performed simulations with *N* = 14, *N* = 20, *N* = 38, and *N* = 66 particles. In agreement with the calculations in the strongly correlated case, there is no noticeable finite size effect for *r*_s_ = 2 at these numbers of particles.

#### 4.3.1 Linear Density Response

**Figure 5:**
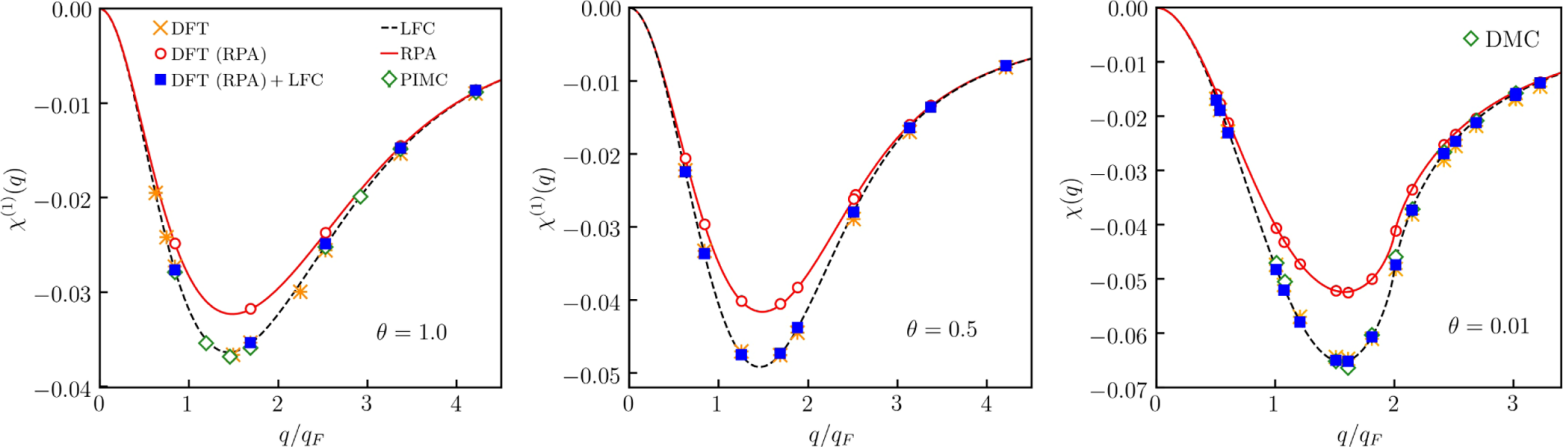
Linear static density response function for *r*_s_ = 2 at θ = 1 (the left panel), at θ = 0.5 (the middle panel), and at θ = 0.01 (the right panel).

In Figure 5, we present results for the linear density response function at *r*_s_ = 2 and compare the KS-DFT data with PIMC results and with χ_LFC_^(1)^ at θ = 1 in the left panel. We observe that the LDA-based results χ̃_LDA_^(1)^ are in good agreement with both the PIMC data and χ_LFC_^(1)^. At θ = 0.5, too, we find good agreement of χ̃_LDA_^(1)^ with χ_LFC_^(1)^ as it can be seen from the middle panel of Figure 5. In the limit of θ → 0, we compare χ̃_LDA_^(1)^ with the DMC data by Moroni et al.^64^ as well as with χ_LFC_^(1)^. Evidently, the LDA-based KS-DFT simulations provide an accurate description of the linear density response function in the strongly degenerate case too. We note that KS-DFT is more accurate in particular for *q* > 2*q*_F_ compared to the previously considered case of *r*_s_ = 6 due to the reduced impact of electronic XC effects at the higher density.

The comparison of χ̃_RPA_^(1)^ with χ_RPA_^(1)^ that is also presented in Figure 5 confirms the high accuracy of the KS-DFT results for the description of the linear response function on the mean-field level across temperature regimes. As a consequence, χ̃_LFC_^(1)^ is in an excellent agreement with χ_LFC_^(1)^ over the entire wavenumber range.

#### 4.3.2 Quadratic Density Response

**Figure 6:**
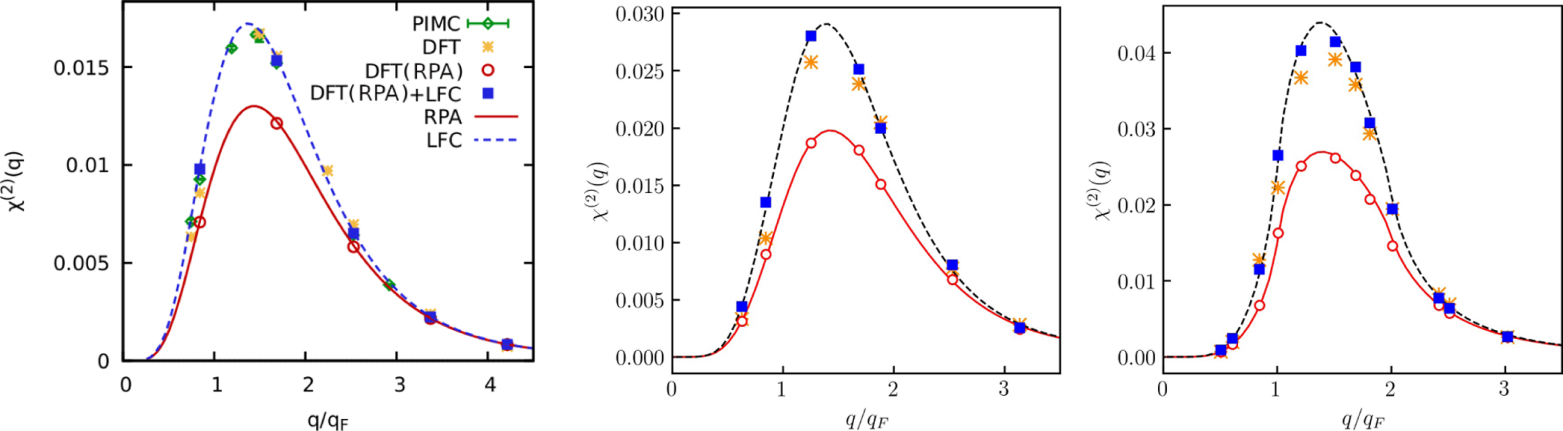
Quadratic static density response function for *r*_s_ = 2 at θ = 1 (the left panel), at θ = 0.5 (the middle panel), and at θ = 0.01 (the right panel).

The results for the quadratic response function at *r*_s_ = 2 are shown in Figure 6. The quadratic response χ̃_LDA_^(2)^ closely reproduces both the PIMC data and χ_LFC_^(2)^ at θ = 1 as it is demonstrated in the left panel of Figure 6. The agreement between χ̃_LDA_^(2)^ and χ_LFC_^(2)^ somewhat deteriorates with the decrease in the temperature from θ = 1 to θ = 0.5 and further to θ = 0.01. This is shown in the middle and right panels of Figure 6. Nevertheless, the LDA, which is an XC functional purely based on the uniform electron gas model, provides an overall impressively accurate description of the quadratic response function at all considered wavenumbers of the perturbation.

Similarly, the discussed cases of the strongly coupled electrons, χ̃_RPA_^(2)^ and χ_RPA_^(2)^ are in close agreement with each other at θ = 1, θ = 0.5, and θ = 0.01. This is a clear illustration of the high accuracy of the theoretical result eq 7 for the mean-field description. Consequently, we find almost the same result using χ̃_LFC_^(2)^ and χ_LFC_^(2)^.

From comparing amplitudes of the quadratic response function in Figure 6 at different temperatures, one can see that the response of the system at the second harmonic becomes stronger upon decreasing the temperature of the electrons. For example, the decrease in the temperature from the partially degenerate case (θ = 1) to the strongly degenerate case (here represented by θ = 0.01) leads to an increase in the maximum value of the quadratic response function by a factor of 2.5. For comparison, the amplitude of the linear response function increases about two times with the decrease in the temperature from θ = 1 to θ = 0.01 at the same conditions.

#### 4.3.3 Cubic Density Response at the First Harmonic

**Figure 7:**
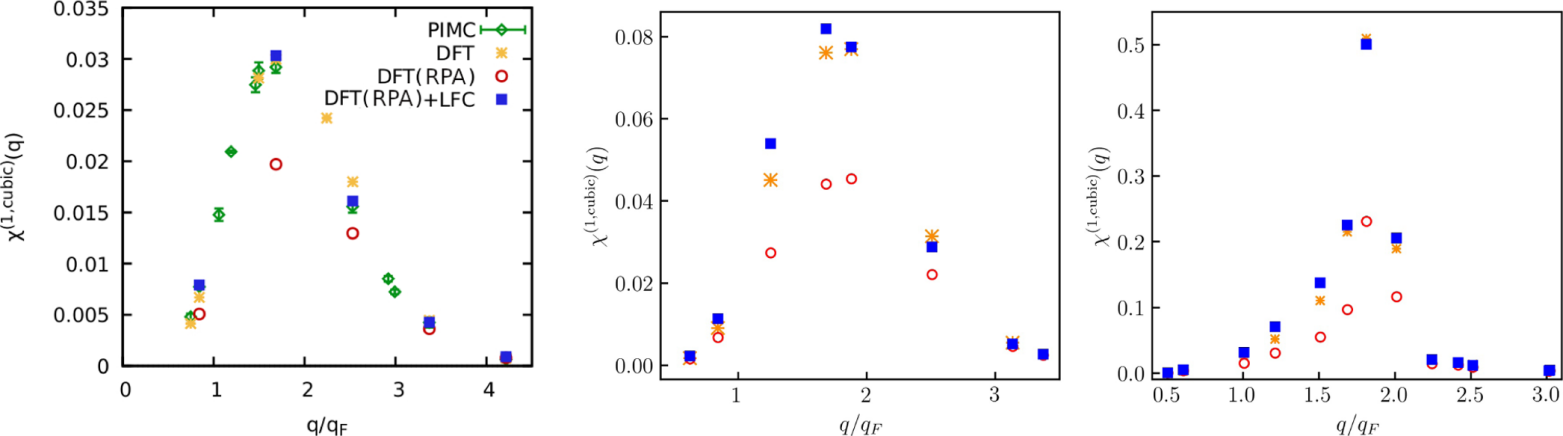
Cubic static density response function at the first harmonic for *r*_s_ = 2 at θ = 1 (the left panel), at θ = 0.5 (the middle panel), and at θ = 0.01 (the right panel).

Let us next investigate the cubic response function at the first harmonic, which is shown in Figure 7. The comparison with the PIMC data at θ = 1 is provided in the left panel of Figure 7. From this comparison, it is evident that the LDA-based KS-DFT calculations are able to provide a very accurate description of the cubic response at *r*_s_ = 2 and θ = 1 as they are in good agreement to both the PIMC data and χ̃_LFC_^(1,cubic)^. This is an indication that the relation (eq 11) holds since χ̃_LFC_^(1,cubic)^ is computed using the KS-DFT data χ̃_RPA_^(1,cubic)^ and the LFC according to eq 18.

Decreasing the electronic temperature leads to a substantial increase in the amplitude of the cubic response function at the first harmonic. This is visible from the comparison of the amplitudes of the cubic response at θ = 1 (left), at θ = 0.5 (middle), and at θ = 0.01 (right) in Figure 7. For example, the maximum of the cubic response function at the first harmonic at θ = 0.01 is about 16 times larger than at θ = 1. The consequences of this remarkable behavior are discussed in more detail in Section 5.

#### 4.3.4 Cubic Density Response at the Third Harmonic

**Figure 8:**
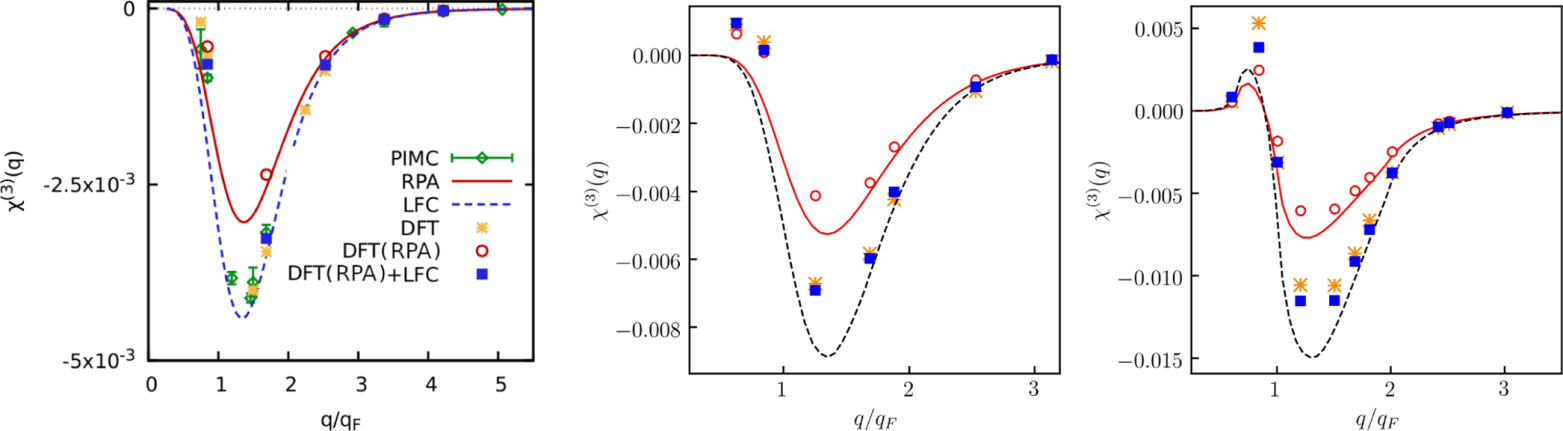
Cubic static density response function at the third harmonic for *r*_s_ = 2 at θ = 1 (the left panel), at θ = 0.5 (the middle panel), and at θ = 0.01 (the right panel).

Finally, we present the results for the cubic response at the third harmonic in Figure 8, and the left panel depicts the comparison with PIMC data at θ = 1. Evidently, the LDA-based calculations χ̃_LDA_^(3)^ are in good agreement with the latter. Next, χ_RPA_^(3)^ overestimates the strength of response compare to χ̃_RPA_^(3)^ at *q* < *q*_F_. In contrast to the response at stronger coupling (*r*_s_ = 6 and θ = 1), at *r*_s_ = 2, we do not see the change in the sign of the cubic response at the third harmonic upon increasing the wavenumber from small *q* < *q*_F_ to large *q* > *q*_F_ values. However, this behavior is restored with a decrease in the temperature to θ = 0.5 as it is clearly visible in the middle panel of Figure 8. Importantly, such behavior at θ = 0.5 is not captured by the analytical results given in eqs 8 and 10. At *q* < 1.5*q*_F_, this leads to a significant disagreement between the analytical result in the mean-field level approximation, eq 8, and the exact result computed using zero XC in the KS-DFT simulations. However, in the limit of strong degeneracy depicted in the right panel of Figure 8, eq 8 again provides the qualitatively correct result by reproducing the change in the sign of the corresponding response function but remains in quantitative disagreement with χ̃_RPA_^(3)^ in the vicinity of the positive maximum and the negative minimum. These disagreements between eq 8 and the DFT (RPA) results could be caused by the approximation of the screening on the linear response level in eq 8 as it was shown by Dornheim et al.^16^

Interestingly, despite the poor performance of eq 8, the agreement between χ̃_LDA_^(3)^ and χ̃_LFC_^(3)^ is rather good at all considered temperature regimes. This is explained by the fact that XC effects are less pronounced compared to the above considered strongly correlated cases.

## 5 Conclusions and Outlook

In this work, we have explored a new methodology for the study of the nonlinear electronic density response based on KS-DFT. As a particular example, we have investigated the free electron gas model and demonstrated that the KS-DFT method is an effective and valuable tool for the investigation of various nonlinear electronic density response functions across temperature regimes. This conclusion is important for parameters where QMC methods experience significant difficulties or fail to converge at all due to the fermion sign problem. This approximately corresponds to θ < 1 and *r*_s_ ≳ 2.^33,36,37^ A particularly effective method to gauge and guide the development of new theoretical approaches is given by the KS-DFT simulation with zero XC functional. This is due to the fact that theoretical models of correlated electrons are built upon mean-field approximations, in combination with the electronic LFC. Therefore, an accurate check of the quality of the analytical results for the nonlinear density response functions in the mean-field approximation is required to build a reliable theory. The presented DFT-based methodology provides such a tool in a wide range of parameters.

As a demonstration of the KS-DFT method-based analysis of the theoretical results, we have considered the quadratic and cubic response functions at different values of the density and degeneracy parameters. First of all, we have confirmed the validity of the analytical results for the quadratic response function (eqs 7 and 9) for partially to strongly degenerate electrons. This confirms and complements the earlier PIMC-based analysis at θ ≥ 1.^16^ Second, it has been shown that the analytical results for the cubic response function at the third harmonic (Eqs 8 and 10) are quantitatively inaccurate at *q* ≲ 1.5 *q*_F_ for all considered values of θ. Moreover, eqs 8 and 10 are qualitatively inadequate at θ = 0.5.

The application of the KS-DFT method to study the cubic response at the first harmonic (as defined in eq 2) has allowed us to observe a change of its characteristics with the decrease in the temperature to θ < 1. We have revealed that the decrease in the temperature from the partially degenerate regime with θ ∼ 1 to the strongly degenerate regime (θ ≪ 1) leads to a significant increase in the maximum of the cubic response at the first harmonic. Let us demonstrate the implication of this finding for electrons at a metallic density, *r*_s_ = 2. Using eqs 2–4 and the data presented in Figures 5–8, one can deduce that, at *r*_s_ = 2, both the quadratic response and the cubic response at the third harmonic remain inferior to the linear density response function if the perturbation amplitude *A* < 1 at all considered θ values and wavenumbers of the perturbation. In contrast, the cubic response function at the first harmonic becomes dominant over the linear response function if *A* > 0.8 at θ = 0.5 and if *A* > 0.36 at θ = 0.01. The applicability of the perturbative analysis requires the smallness of the higher order correction compared to the first order term in eq 2. Therefore, at θ ≪ 1, not the quadratic response but the cubic response at the first harmonic leads to the strongest restriction on the applicability of the nonlinear density response theory of free electrons with respect to the perturbation amplitude.

Another important finding is that the LDA XC functional provides a remarkably accurate description of the linear and nonlinear density response at metallic density, *r*_s_ = 2, across the entire considered temperature range. This strongly indicates that our new KS-DFT-based approach constitutes a reliable tool for the investigation of the nonlinear density response both at ambient conditions and in the WDM regime. At stronger coupling parameters, *r*_s_ = 6 and *r*_s_ = 5, the LDA XC functional-based KS-DFT calculations of the correlated electron gas provide qualitatively correct behavior but ought to be considered with caution from a quantitative point of view.

We conclude this study by pointing out that the present methodology is very general and can be directly applied to arbitrary materials; the only approximation is given the choice of the XC-functional. In particular, simulations including ions are much more problematic and computationally expensive for the QMC methods compared to the considered case of a free electron gas model. Therefore, the KS-DFT method is particularly valuable for the multi-component systems. This proof of concept of the capability of KS-DFT for the estimation of the NLRT of an electron gas is thus a pivotal first step before extending our considerations to real materials.

## Figures and Tables

**Figure 9:**
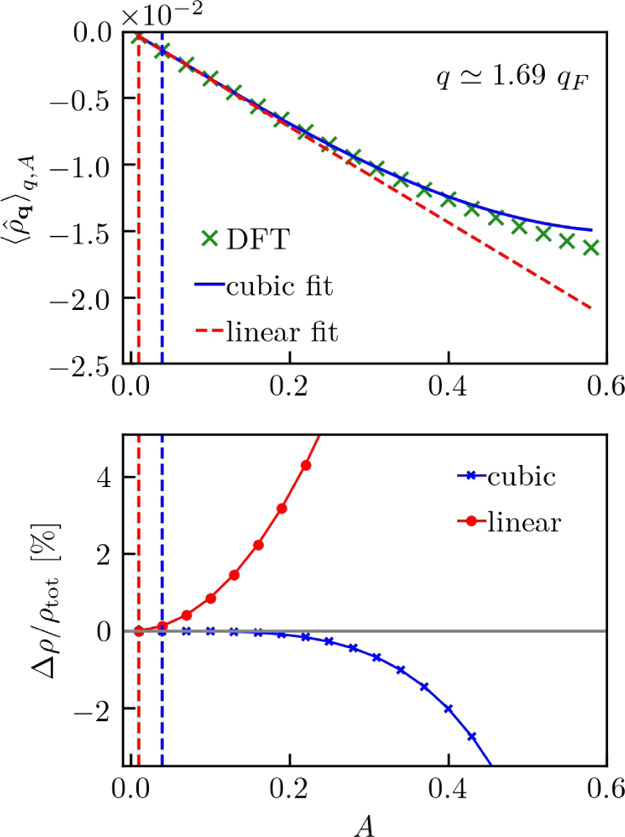
Density perturbation component ⟨ρ̂_**k**_⟩_*q*,*A*_ at *k* = *q* is used to find the linear density response and the cubic density response at the first harmonic at *r*_s_ = 2 and θ = 1.

**Figure 10:**
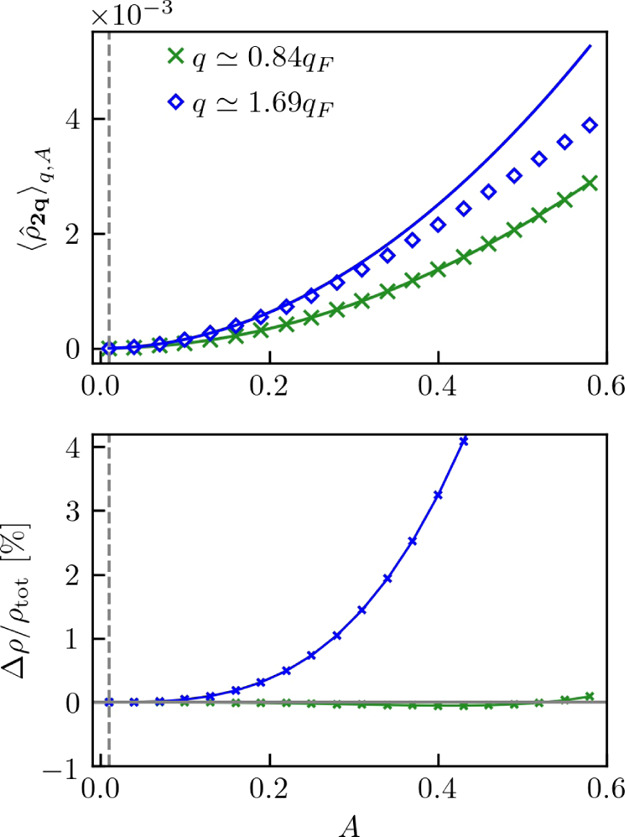
Density perturbation component ⟨ρ̂_**k**_⟩_*q*,*A*_ at *k* = 2*q* is used to find the quadratic density response at *r*_s_ = 2 and θ = 1.

**Figure 11:**
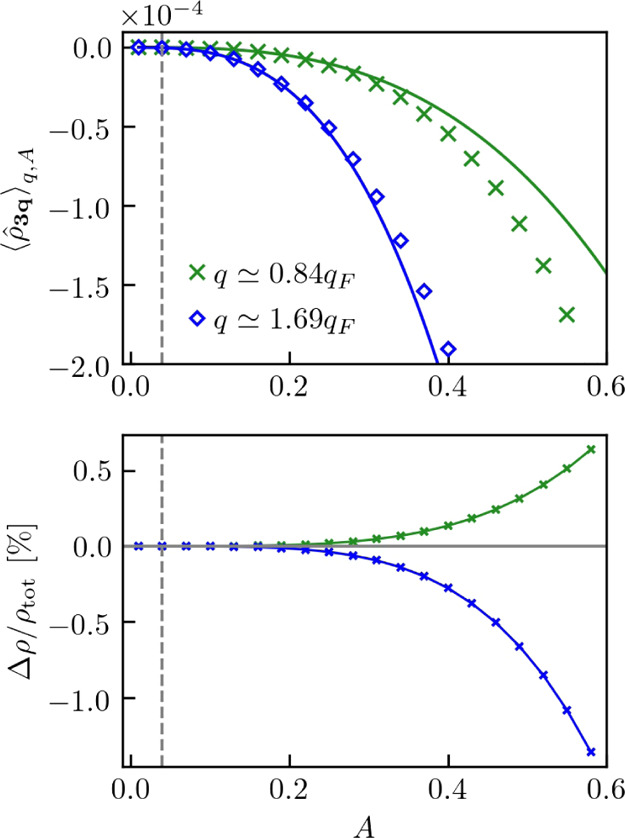
Density perturbation component ⟨ρ̂_**k**_⟩_*q*,*A*_ at *k* = 3*q* is used to find the cubic density response at *r*_s_ = 2 and θ = 1.

## Appendix

### Illustration of the Extraction of Response Functions from the Density Perturbation

Here, we illustrate the extraction of the density response functions from the density perturbation components in Fourier space ⟨ρ̂_**k**_⟩_*q*,*A*_. As an example, we consider *r*_s_ = 2 and θ = 1. The results are computed by performing KS-DFT simulations of *N* = 14 electrons in the main cell using the LDA XC functional.

In the top panel of Figure 9, we show the density perturbation at the first harmonic (*k* = *q*) with the perturbation wavenumber *q* ≃ 1.69*q*_F_. The results for eq 2 are given by the solid line, where χ^(1)^ and χ^(1,cubic)^ in eq 2 are found using the first two points (as indicated by the vertical lines). This corresponds to a cubic fit which takes into account both the linear density response and the cubic density response at the first harmonic. In this way, we extract data for these response functions. For comparison, we also show the linear response result neglecting the cubic contribution (dashed line). We see that the inclusion of the cubic contribution allows us to extend the description of the density response beyond *A* = 0.16 up to *A* = 0.4 (with an error less than 2 %). The corresponding values of the relative difference between the simulation results and the data computed using eq 2 are depicted in the bottom panel of Figure 9, where the difference is divided by the value of the total density perturbation ρ_tot_. The latter allows us to estimate the resulting error in the description of the total density.

Similar analysis is presented for the density response at the second harmonic of the original perturbation (*k* = 2*q*), as shown in Figure 10, and the top panel depicts results for the perturbation wavenumbers *q* ≃ 1.69*q*_F_ and *q* ≃ 0.84*q*_F_. The KS-DFT results are computed using the perturbation amplitudes in the range 0.01 ≤ *A* ≤ 0.6. The solid lines show the fit according to eq 3 based only on the first data point at *A* = 0.01 (indicated by the vertical line). This allows one to find the value of the quadratic response. The corresponding values of the relative difference between the KS-DFT data and the result of the fit based on eq 3 are given in the bottom panel of Figure 10.

Finally, we show the results for the density response at the third harmonic (*k* = 3*q*) in the top panel of Figure 11. In this case, the KS-DFT simulations are performed for 0.01 ≤ *A* ≤ 0.6. The cubic response function at the third harmonic is found using the data for ⟨ρ̂_**3q**_⟩_*q*,*A*_ at *A* = 0.04. The values of the relative difference between the KS-DFT data and the results computed using eq 4 are provided in the bottom panel of Figure 11.

All KS-DFT-based results for the linear and nonlinear density response that have been presented in this work for different wavenumbers, densities, and temperatures have been obtained by performing similar analysis to those shown in Figures 9–11 but involving a reduced number of *A* values. The specific ranges of the considered *A* values are given in Section 3.

## Notes

The data that support the findings of this study are available from the corresponding author upon reasonable request.
